# Characterization of the Heme Pocket Structure and Ligand Binding Kinetics of Non-symbiotic Hemoglobins from the Model Legume *Lotus japonicus*

**DOI:** 10.3389/fpls.2017.00407

**Published:** 2017-04-04

**Authors:** Laura Calvo-Begueria, Bert Cuypers, Sabine Van Doorslaer, Stefania Abbruzzetti, Stefano Bruno, Herald Berghmans, Sylvia Dewilde, Javier Ramos, Cristiano Viappiani, Manuel Becana

**Affiliations:** ^1^Departamento de Nutrición Vegetal, Estación Experimental de Aula Dei, Consejo Superior de Investigaciones CientíficasZaragoza, Spain; ^2^Department of Physics, University of AntwerpAntwerp, Belgium; ^3^Dipartimento di Bioscienze, Università degli Studi di ParmaParma, Italy; ^4^NEST, Istituto Nanoscienze, Consiglio Nazionale delle RicerchePisa, Italy; ^5^Dipartimento di Farmacia, Università degli Studi di ParmaParma, Italy; ^6^Department of Biomedical Sciences, University of AntwerpAntwerp, Belgium; ^7^Dipartimento di Fisica e Scienze della Terra, Università degli Studi di ParmaParma, Italy

**Keywords:** heme cavity, ligand binding, nitric oxide dioxygenase, non-symbiotic hemoglobins, *Lotus japonicus*

## Abstract

Plant hemoglobins (Hbs) are found in nodules of legumes and actinorhizal plants but also in non-symbiotic organs of monocots and dicots. Non-symbiotic Hbs (nsHbs) have been classified into two phylogenetic groups. Class 1 nsHbs show an extremely high O_2_ affinity and are induced by hypoxia and nitric oxide (NO), whereas class 2 nsHbs have moderate O_2_ affinity and are induced by cold and cytokinins. The functions of nsHbs are still unclear, but some of them rely on the capacity of hemes to bind diatomic ligands and catalyze the NO dioxygenase (NOD) reaction (oxyferrous Hb + NO → ferric Hb + nitrate). Moreover, NO may nitrosylate Cys residues of proteins. It is therefore important to determine the ligand binding properties of the hemes and the role of Cys residues. Here, we have addressed these issues with the two class 1 nsHbs (LjGlb1-1 and LjGlb1-2) and the single class 2 nsHb (LjGlb2) of *Lotus japonicus*, which is a model legume used to facilitate the transfer of genetic and biochemical information into crops. We have employed carbon monoxide (CO) as a model ligand and resonance Raman, laser flash photolysis, and stopped-flow spectroscopies to unveil major differences in the heme environments and ligand binding kinetics of the three proteins, which suggest non-redundant functions. In the deoxyferrous state, LjGlb1-1 is partially hexacoordinate, whereas LjGlb1-2 shows complete hexacoordination (behaving like class 2 nsHbs) and LjGlb2 is mostly pentacoordinate (unlike other class 2 nsHbs). LjGlb1-1 binds CO very strongly by stabilizing it through hydrogen bonding, but LjGlb1-2 and LjGlb2 show lower CO stabilization. The changes in CO stabilization would explain the different affinities of the three proteins for gaseous ligands. These affinities are determined by the dissociation rates and follow the order LjGlb1-1 > LjGlb1-2 > LjGlb2. Mutations LjGlb1-1 C78S and LjGlb1-2 C79S caused important alterations in protein dynamics and stability, indicating a structural role of those Cys residues, whereas mutation LjGlb1-1 C8S had a smaller effect. The three proteins and their mutant derivatives exhibited similarly high rates of NO consumption, which were due to NOD activity of the hemes and not to nitrosylation of Cys residues.

## Introduction

The first plant hemoglobins (Hbs) were discovered in the root nodules of legumes and accordingly designated leghemoglobins (Appleby, 1984). The discovery of Hbs was subsequently extended not only to the nodules of *Parasponia* and actinorhizal plants (Tjepkema et al., 1986; Bogusz et al., 1988), but also to non-symbiotic tissues of monocots (Taylor et al., 1994; Arredondo-Peter et al., 1997), legumes (Andersson et al., 1996), and *Arabidopsis thaliana* (Trevaskis et al., 1997). Phylogenetic analyses showed that these non-symbiotic Hbs (nsHbs) belong to two distinct clades, termed class 1 and class 2 (Smagghe et al., 2009). Class 1 nsHbs have an extremely high O_2_ affinity and are induced by hypoxia (Trevaskis et al., 1997; Smagghe et al., 2009) and by exposure to nitrate, nitrite, or nitric oxide (NO) (Sasakura et al., 2006). These proteins may play a role in plant survival by increasing the energy status of the cells under hypoxic conditions (Igamberdiev and Hill, 2004). The underlying molecular mechanism is thought to be the Hb/NO cycle, in which the NO dioxygenase (NOD) activity of Hb plays a critical role (Igamberdiev and Hill, 2004). In this reaction, the oxyferrous Hb dioxygenates NO to yield nitrate and ferric Hb. The NOD activities of a few class 1 nsHbs, including *A. thaliana* Hb1 (AtGlb1), have been measured *in vitro* (Perazzolli et al., 2004; Igamberdiev et al., 2006; Smagghe et al., 2008). However, AtGlb1 may be also nitrosylated by NO on Cys residues and this might affect its function (Perazzolli et al., 2004). Class 2 nsHbs have a moderate O_2_ affinity and are induced by low temperature and cytokinins but not by hypoxia (Hunt et al., 2001).

At the molecular level, the control of hemeprotein function is tightly coupled to the structure and ligand-binding dynamics of the heme pocket. For plant Hbs, early studies on the heme properties have been focused on leghemoglobins (Appleby et al., 1976; Rousseau et al., 1983), although more recently some information has become available about the hemes of nsHbs (Das et al., 1999; Ioanitescu et al., 2005; Abbruzzetti et al., 2007; Bruno et al., 2007a). Class 1 and class 2 Hbs and some animal globins, such as neuroglobin and cytoglobin, are hexacoordinate because, in the absence of exogenous ligands, they have the fifth (proximal) and sixth (distal) positions of the heme iron coordinated to His residues; in contrast, leghemoglobins and mammalian Hb and myoglobin (Mb) are pentacoordinate because the fifth position of the heme iron is coordinated to a His residue but the sixth position is open for ligand binding (Kakar et al., 2010). Interestingly, a system of hydrophobic cavities, capable of transiently stocking reactants and/or products, was proposed to be central to sustain the turnover of NOD activity in class 1 nsHbs (Spyrakis et al., 2011; Abbruzzetti et al., 2013), as may occur for Mb (Brunori, 2001) and neuroglobin (Uzan et al., 2004; Brunori et al., 2005).

Legumes are important in agriculture for two main reasons: they are a source of protein for animal and human nutrition, and they can establish nitrogen-fixing symbioses with soil rhizobia, allowing to minimize the supply of contaminant and costly nitrogen fertilizers. Besides leghemoglobins, whose expression is restricted to nodules, legumes contain nsHbs in leaves, roots, and nodules (Andersson et al., 1996; Bustos-Sanmamed et al., 2011). In fact, some of these nsHbs have important functions in the onset of symbiosis (Fukudome et al., 2016) and exhibit high expression in nodules compared with other plant organs (Bustos-Sanmamed et al., 2011). In this work, we have studied the nsHbs of *Lotus japonicus*, a model legume for classical and molecular genetics (Handberg and Stougaard, 1992). We have selected *L. japonicus* instead of *A. thaliana* as plant material because the information gained about the nsHbs of the former species will help define their role in symbiosis and will facilitate translational genomics to crop legumes. More specifically, the use of *L. japonicus* has allowed us to compare herein the biochemical properties of two class 1 nsHbs (LjGlb1-1 and LjGlb1-2) and a class 2 nsHb (LjGlb2) that greatly differ in their expression profiles in plant tissues (Bustos-Sanmamed et al., 2011) and in their O_2_ affinities (Sainz et al., 2013). These previous results from one of our laboratories prompted us to investigate the heme environment properties of LjGlbs, as well as the contribution of their Cys residues to protein stability and ligand binding kinetics. To accomplish these objectives, we have performed a detailed spectroscopic study of the wild-type and the mutated proteins and have measured their NOD activities.

## Materials and Methods

### Protein Purification and Identification of Disulfide Bond

The three nsHbs of *L. japonicus* were cloned into the Champion pET200/D-TOPO expression vector (Invitrogen) and expressed with an N-terminal poly-His tag in *Escherichia coli* BL21 Star (DE3) cells (Invitrogen) or C41(DE3) cells (Lucigen; Middleton, MI, USA) following conventional protocols (Sainz et al., 2013). The mutated versions of LjGlb1-1 C8S, LjGlb1-1 C78S, LjGlb1-2 C79S, and LjGlb2 C65S were obtained by PCR-based single-site substitutions using appropriate primers (Mutagenex; Somerset, NJ, USA). The DNA constructs were entirely sequenced and the amino acid substitutions (Supplementary Figure S1) were verified by matrix-assisted laser desorption and ionization time-of-flight (MALDI-TOF) mass spectrometry analysis of the trypsinized protein using a 4800 TOF/TOF instrument (AB Sciex; Framingham, MA, USA). The proteins were purified using ammonium sulfate fractionation, metal-affinity chromatography, and anion-exchange chromatography, as reported earlier (Sainz et al., 2013). Purification of LjGlb1-1 was carried out in the presence of 200 mM NaCl to avoid precipitation of the dimeric form. The presence of LjGlb1-1 homodimer was demonstrated by fast protein liquid chromatography (FPLC) and mass spectrometry. For FPLC, the purified protein (10 mg) was loaded on a gel filtration column (Superdex 200 HR 10/30) coupled to an ÄKTA FPLC chromatography system (GE Healthcare Life Sciences), and was eluted with 50 mM potassium phosphate (pH 7.0) containing 150 mM NaCl at a flow rate of 0.5 ml min^-1^. The void volume was calculated with dextran blue (0.1 mg ml^-1^) and the column was calibrated with cytochrome *c* (12.4 kDa), Mb (17 kDa), ovalbumin (44.3 kDa), and bovine serum albumin (66 kDa). For molecular mass determinations, the purified protein was diluted 1:50 with 0.2% trifluoroacetic acid and analyzed by MALDI-TOF mass spectrometry. Calibration was performed with a mixture of albumin, trypsinogen, and protein A (mass range between 22,306 and 66,431 Da), and the accuracy was ±50 Da at 40 kDa.

### Resonance Raman Spectroscopy

Resonance Raman (RR) spectra were acquired using a Dilor XY-800 spectrometer in low-dispersion mode using a liquid N_2_-cooled CCD detector. The excitation source was a Spectra Physics (Mountain View, CA, USA) BeamLok 2060 Kr^+^ laser operating at 413.1 nm. The spectra were recorded at room temperature and the protein solutions were magnetically stirred at 500 rpm in order to avoid local heating and photochemical decomposition. The slit width used during the experiments was 200 μm. In general, 12–15 spectra were acquired with an integration time of 150–180 s each. Spikes due to cosmic rays were removed by omitting the highest and lowest data points for each frequency and by averaging the remaining values. Typical sample concentrations were in the order of 40–60 μM.

### Ligand Binding Kinetics

Laser flash photolysis (LFP) experiments were performed at 20°C using a laser photolysis system (Edinburgh Instruments LP920, UK) equipped with a frequency-doubled, Q-switched Nd:YAG laser (Quanta-Ray, Spectra Physics). The carbon monoxide (CO)–ferrous Hb complexes were prepared in sealed 4 × 10 mm quartz cuvettes with 1 ml of 100 mM potassium phosphate buffer (pH 7.0) containing 1 mM EDTA. In the case of LjGlb1-1 this buffer was supplemented with 200 mM NaCl to improve protein stability. The buffer was equilibrated with mixtures of CO and N_2_ in different ratios to obtain CO concentrations of 50–800 μM by using a gas mixer (High-Tech System; Bronkhorst, The Netherlands). Saturated sodium dithionite solution (10 μl) was added and the protein was injected to a final concentration of ∼4 μM. Formation of the CO–ferrous Hb complex was verified by UV/visible absorption spectroscopy. Recombination of the photo-dissociated CO-ligand was monitored at 417 nm.

### Stopped Flow Experiments

Stopped flow measurements were performed in 100 mM degassed potassium phosphate buffer (pH 7.0) and 1 mM EDTA at 20°C, by using a thermostated stopped flow apparatus (Applied Photophysics; Salisbury, UK). Sodium dithionite was added to both the protein solution and the CO solutions to a final concentration of 10 mM. Measurements were carried out during 2 s at 414 nm with 4 μM of protein solution that was mixed with different CO concentrations. Analysis was performed by using Origin software.

### Nitric Oxide Dioxygenase Activities

NOD activities were assayed by following the disappearance of NO (Igamberdiev et al., 2006) with a selective electrode (ISO-NOP) coupled to a free radical analyzer (TBR4100), both from World Precision Instruments (Sarasota, FL, USA). The proteins were converted to the oxyferrous form by reduction with a trace of dithionite and rapid oxygenation through NAP-5 mini-columns (GE Healthcare Life Sciences). NOD activities were measured with diethylamine NONOate (DEA) and *S*-nitrosoglutathione (GSNO) as NO donors. DEA was purchased from Sigma and was freshly prepared for each assay. GSNO was synthesized by mixing 1 mM of acidified nitrite and 1 mM glutathione; the solution was rapidly neutralized and GSNO was quantified, aliquoted, and stored at -80°C protected from light (Smagghe et al., 2008). Concentrations of DEA and GSNO were standardized just prior to the assays by using extinction coefficients of 8 mM^-1^ cm^-1^ at 250 nm and 0.85 mM^-1^ cm^-1^ at 335 nm, respectively.

For the assay, DEA (20 μM) or GSNO (1 mM) was added to a final volume of ∼4 ml of 50 mM potassium phosphate buffer (pH 7.5) containing 50 μM diethylenetriaminepentaacetic acid. The solution was gently stirred at 24°C until NO concentration became stable (∼6 μM with DEA and ∼2 μM with GSNO after ∼4 min). The oxyferrous Hb (1 μM; 30–60 μl) was added, while stirring, so that the final volume of the reaction mixture was exactly 4 ml, and the decrease in NO concentration was measured. The time between the preparation of oxyferrous Hbs and the assays of NOD activity was always <5 min. The corresponding ferric globins lacked NOD activity and were employed as negative controls. The NO electrode was calibrated for each set of measurements by following the manufacturer’s instructions.

## Results

### Purification of nsHbs and Identification of Disulfide Bond

Recombinant LjGlbs, and the mutant derivatives LjGlb1-1 C8S, LjGlb1-1 C78S, and LjGlb1-2 C79S, were highly purified and the protein preparations usually exhibited Soret/A_280_ ratios >2.8. Unfortunately, we were unable to produce the LjGlb2 C65S at enough yield for kinetic and structural studies because of the instability of the protein. We found that LjGlb1-2 and LjGlb2 are monomeric proteins. However, LjGlb1-1 was present both as a monomer and dimer when purified in the presence of 200 mM NaCl, whereas only the monomer was found when the salt was omitted during purification. The homodimer was formed by a disulfide bond, as revealed by FPLC and mass spectrometry analysis in the absence and presence of dithiothreitol (Supplementary Figure S2). The disulfide bridge involves Cys8 because the LjGlb1-1 C78S mutant is still able to form a dimer that disappears upon addition of dithiothreitol (data not shown). Interestingly, barley Hb1 is a homodimer having a disulfide bond through its unique residue and hence the protein is stable without salt (Duff et al., 1997; Bykova et al., 2006), whereas rice Hb1 is also a homodimer but does not appear to form disulfide bridges (Goodman and Hargrove, 2001). We found that the dimer of LjGlb1-1 precipitated if salt was omitted during purification and the spectroscopic studies of the wild-type LjGlb1-1 and its mutated forms were therefore performed in buffer supplemented with 200 mM NaCl.

### Resonance Raman Spectroscopy

Earlier work has shown that RR spectroscopy is most useful to identify different oxidation and ligation states of the globins and to study in detail the stabilization of heme ligands by the amino acid residues in the heme pocket (Hu et al., 1996). Accordingly, we have used RR to compare the heme environments in the wild-type and mutant LjGlbs.

**FIGURE 1:**
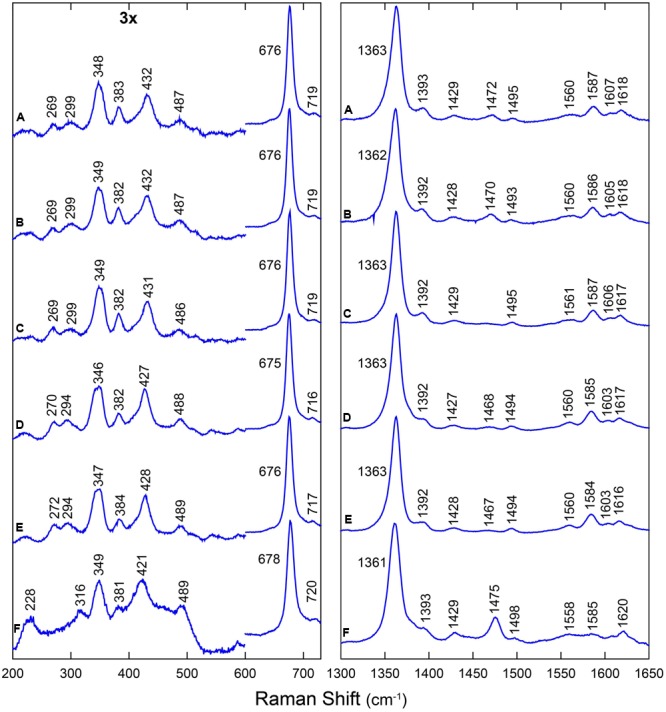
**RR spectra of LjGlbs in the deoxyferrous state.** The spectra correspond to **(A)** LjGlb1-1, **(B)** LjGlb1-1 C8S, **(C)** LjGlb1-1 C78S, **(D)** LjGlb1-2, **(E)** LjGlb1-2 C79S, and **(F)** LjGlb2. All the spectra were recorded with a laser power of 12 mW.

The RR spectra were obtained in the high-frequency region (1300–1650 cm^-1^), which contains marker bands for the oxidation, coordination, and spin state of the heme iron, as well as in the low-frequency region (200–700 cm^-1^), which contains several in-plane and out-of-plane vibrational modes of the heme. **Figure 1** shows the RR spectra of all the wild-type proteins and their mutated versions in their deoxyferrous form. All RR spectra show marker bands ν_4_ at 1361–1363 cm^-1^ and ν_3_ at 1493–1496 cm^-1^, which are characteristic for a hexacoordinate low-spin (6cLS) ferrous form. Additionally, a second ν_3_ band is seen at 1467–1475 cm^-1^, which indicates the presence of a pentacoordinate high-spin (5cHS) ferrous form. This band has a substantially larger intensity for LjGlb2 relative to LjGlb1-1 and LjGlb1-2. Although the intensities of the two ν_3_ marker bands seem to be similar for LjGlb1-1 and LjGlb1-2, the population of the pentacoordinate (5c) ferrous heme is nevertheless smaller than that of the hexacoordinate (6c) ferrous heme. Indeed, the intrinsic intensity of the ν_3_ marker band of 5c heme is much higher than that of 6c heme (Das et al., 1999), which makes it difficult to get accurate relative populations out of the RR spectra. This is confirmed by the optical absorption spectra that were shown in earlier work (Sainz et al., 2013), which indicated that in the ferrous forms of LjGlb1-1 and LjGlb1-2, a mixture between 5cHS and 6cLS is observed, with the latter being the dominant species. On the contrary, in the ferrous form of LjGlb2, the 5cHS species prevails over the 6cLS species, in line with earlier absorption measurements (Sainz et al., 2013). Taken together, the RR and UV/visible spectra reveal that, for LjGlb1-1 and LjGlb1-2, a 5cHS form is present as a minor fraction, whereas this constitutes the most abundant fraction in LjGlb2. The equilibrium between the 5cHS and 6cLS species, evidenced by the spectroscopic data, is confirmed by the distal His binding constants reported in **Table 1**. The high-frequency region of the RR spectra of the deoxyferrous form of both LjGlb1-1 and LjGlb1-2 is similar to that found for barley Hb1 (Das et al., 1999), tomato Hb1 (Ioanitescu et al., 2005), and AtGlb2 (Bruno et al., 2007a), which all have a bis-histidyl coordination of the heme. This is in contrast with LjGlb2, which has a mixed 5cHS–6cLS state in the ferrous form and is more similar to AtGlb1 (Bruno et al., 2007a). Mutation of Cys to Ser alters the relative intensity ratio of the ν_3_ marker bands for LjGlb1-1 (**Figures 1A–C**). More specifically, the relative fraction of 5cHS increases for LjGlb1-1 C8S and decreases for LjGlb1-1 C78S when compared to the wild-type protein. Overlay of the RR spectra of LjGlb1-2 and LjGlb1-2 C79S shows a small decrease in the 5cHS contribution in the mutant (**Figures 1D,E**).

**Table 1:** Rate constants of LjGlbs and their mutated derivatives.

Globin	*k*_on,CO_^a^ (μM^-1^ s^-1^)	*k*_on,H_^b^ (s^-1^)	*k*_off,H_^a,b^ (s^-1^)	*K*_H_^c^	*F*_H_^d^	*F*_gem_^e^	*k*_gem_^f^ (×10^7^ s^-1^)
LjGlb1-1	1.2	67	27	2.48	0.71	0.09	9.2
LjGlb1-1 C8S	1.6	111	57	1.95	0.66	0.04	6.8
LjGlb1-1 C78S	1.7	288	5.1	56.8	0.98	0.13	6.2
LjGlb1-2	29	1484	88	16.9	0.94	0.27	7.6
LjGlb1-2 C79S	67	3501/1051	84/5.8	41.7/181	0.98/0.99	0.46	8.8
LjGlb2^g^	3.79 (70%) 10.71 (30%)	0.033/4.4	0.15/20	0.22	0.18	0.15	5.7

*^a^Determined by LFP. ^b^Determined by stopped flow. ^c^Equilibrium constant for His binding *K*_*H*_ = *k*_*on,H*_/*k*_*off,H*_. ^d^*F*_*H*_ = *K*_*H*_/(1 + *K*_*H*_), fraction of bis*-*histidyl 6c species in ferrous proteins at equilibrium. ^e^*F*_*gem*_, fractional amplitude of geminate rebinding. ^f^*k*_*gem*_, rate constant for geminate rebinding. ^g^For LjGlb2, the hexacoordinated fraction *F*_*H*_ is calculated from the absorption spectrum and is consistent with the amplitude of binding to the 5c species in stopped flow. Because *K*_*H*_ = *F*_*H*_/(1 -*F*_*H*_), from *F*_*H*_ = 0.18 we obtain *K*_*H*_ = 0.22. There are two *k*_*off,H*_ values (0.15 and 20 s^-*1*^). Assuming the same equilibrium constant, we can calculate two values for *k*_*on,H*_ = *K*_*H*_. *k*_*off,H*_ (0.033 and 4.4 s^-*1*^, respectively).*

The low-frequency region (200–730 cm^-1^) of the RR spectra contains a number of bending modes from the vinyl and propionate substituents of the heme group. The propionate bending mode [δ(C_β_ - C_c_ - C_d_)] appears at 382–384 cm^-1^. For comparison, this mode is found at 380 cm^-1^ in ferrous barley Hb (Das et al., 1999) and tomato Hb (Ioanitescu et al., 2005). It is possible to use this mode to quantify the strength of the interaction between the heme propionate groups and nearby amino acid residues; a higher Raman shift for the propionate bending mode indicates a stronger interaction (Cerda-Colon et al., 1998). This interaction seems to be similar for all studied LjGlbs. When compared with ferrous barley and tomato Hbs, the interaction seems stronger for the LjGlbs. The vinyl bending modes [δ(C_β_ - C_a_ - C_b_)] are seen as a single line at 428–432 cm^-1^ (LjGlb1-1 and LjGlb1-2) and 422 cm^-1^ (LjGlb2). This indicates a stronger interaction of the vinyl group with surrounding amino acid residues for both class 1 Hbs. In barley Hb the vinyl bending modes were found at 425 cm^-1^. The overlap of both bending modes of the vinyl groups indicates a relaxed heme configuration. The γ_7_ pyrrole bending mode, which is associated with a heme out-of-plane distortion, is only visible for LjGlb2 at 316 cm^-1^ (**Figure 1F**), in line with the relative high fraction of 5cHS heme in this protein. Both LjGlb1-1 and LjGlb1-2 lack out-of-plane modes γ_6_, γ_7_, γ_12_, and γ_21_, which is typical for a bis-histidyl coordination. This indicates, together with the overlap of both bending modes of the vinyl groups, that for the class 1 nsHbs, the heme is in a relaxed state with the heme iron almost completely in the porphyrin plane. Finally, the ν_Fe-His_ stretching mode (228 cm^-1^) is only visible for LjGlb2 in the deoxyferrous state. This is in agreement with the presence of the 5cHS form, since the Fe-His stretching mode is generally not observed for bis-histidyl coordinated globins. The value of ν_Fe-His_ of LjGlb2 is somewhat higher than that observed for barley Hb (219 cm^-1^) but still typical for globins (Das et al., 1999).

**FIGURE 2:**
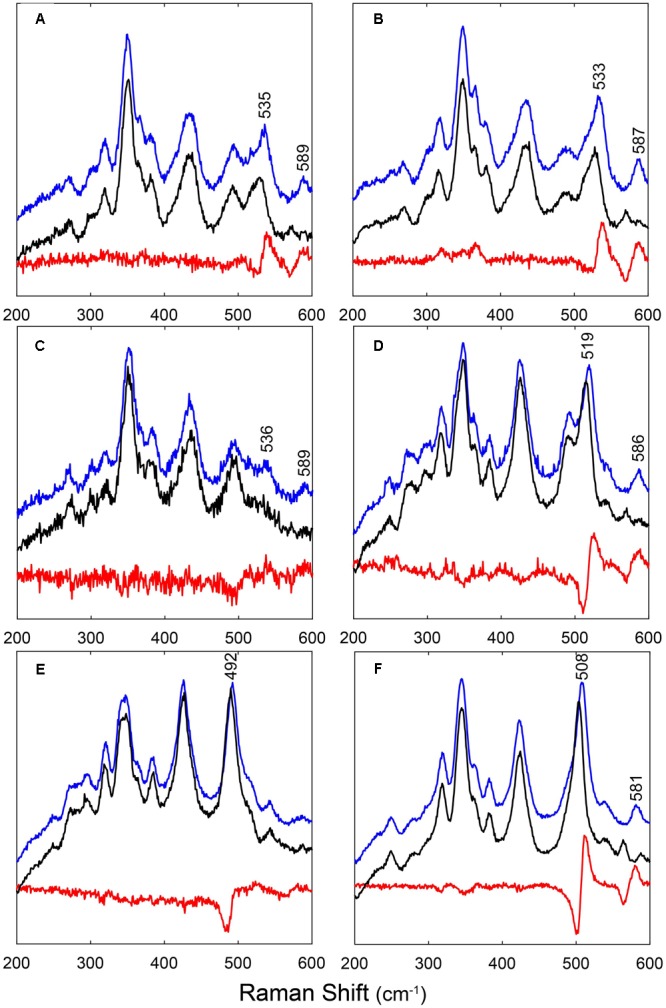
**RR spectra of LjGlbs and of their mutant derivatives in the ferrous CO-ligated form.** The figure shows the spectra of **(A)** LjGlb1-1, **(B)** LjGlb1-1 C8S, **(C)** LjGlb1-1 C78S, **(D)** LjGlb1-2, **(E)** LjGlb1-2 C79S, and **(F)** LjGlb2. The upper (*blue*), middle (*black*), and lower (*red*) spectra correspond to the ^12^CO form, the ^13^CO form, and the difference spectra, respectively. All spectra were recorded with a laser power of 1 mW.

**Figure 2** shows the RR spectra of the ferrous ^12/13^CO-ligated forms of the globins and their mutated forms. In addition, Supplementary Figure S3 includes a comparison of the RR spectra of the ferrous CO-ligated forms of all proteins recorded with low (1 mW) and high (35–165 mW) laser power. Upon increasing the laser power, partial photolysis occurs which is apparent from the shift of the ν_4_ frequency from the CO-ligated (∼1375 cm^-1^) to the ferrous (∼1362 cm^-1^) state. Because of this photolysis, a general decrease in intensity of the Fe-CO stretching modes (ν_Fe-CO_) is expected. We clearly see a spectral change in the 450-600 cm^-1^ region, where the Fe-CO stretching modes occur. Further assignment of these bands is corroborated by comparing the RR spectra of the ^12^CO- and ^13^CO-ligated globins (**Figure 2**). The use of ^13^CO induces a downward shift in the ν_Fe-CO_ modes of ∼3 cm^-1^, whereas the Fe-CO bending mode (δ_Fe-CO_) mode shifts from 582–589 cm^-1^ to 564–569 cm^-1^. This can be better seen in the difference spectrum (^12^CO–^13^CO) (**Figure 2**; *red spectra*).

The ν_Fe-CO_ modes are sensitive to interactions of the CO ligand with nearby amino acid residues. Whereas Fe-CO stretching modes around 490–495 cm^-1^ indicate an open heme pocket in which the CO interacts only weakly with the surrounding amino acids, higher ν_Fe-CO_ modes are due to a closed heme pocket in which a positively charged amino acid residue is stabilizing the CO group (Spiro and Wasbotten, 2005; Bruno et al., 2007a). In general, the higher the mode, the stronger the CO ligand will be hydrogen bonded and interact with the positively charged residue. For LjGlb1-1 and its C8S and C78S mutants, the ν_Fe-CO_ modes were found at ∼533–536 cm^-1^, with δ_Fe-CO_ at 587–589 cm^-1^ (**Figures 2A–C** and Supplementary Figure S3a–c). This indicates a very strong interaction of the CO ligand, which is not affected by the mutations. In contrast, the ν_Fe-CO_ mode is at 519 cm^-1^ for wild-type LjGlb1-2, but at 492 cm^-1^ for its C79S mutant (**Figures 2D,E** and Supplementary Figure S3d,e). In this case, the mutation induces a switch from a closed to an open heme pocket. Consistent with this, the Fe-CO bending mode is clearly visible for the closed configuration (∼586 cm^-1^) of the wild-type protein, but it is hardly observed for the open heme pocket of the mutant (**Figure 2E**). Finally, the ν_Fe-CO_ mode of LjGlb2 at 508 cm^-1^ (**Figure 2F** and Supplementary Figure S3f) is similar to that observed for CO-ligated vertebrate Mbs, where the CO stabilization occurs through the His(E7) residue. The interaction of bound CO with the surrounding amino acid residues is thus weaker in class 2 than in class 1 Hbs, in line with the occurrence of a dominant fraction of 5cHS form in the deoxyferrous state of LjGlb2 (**Figure 1F**).

### Ligand Binding Kinetics

**FIGURE 3:**
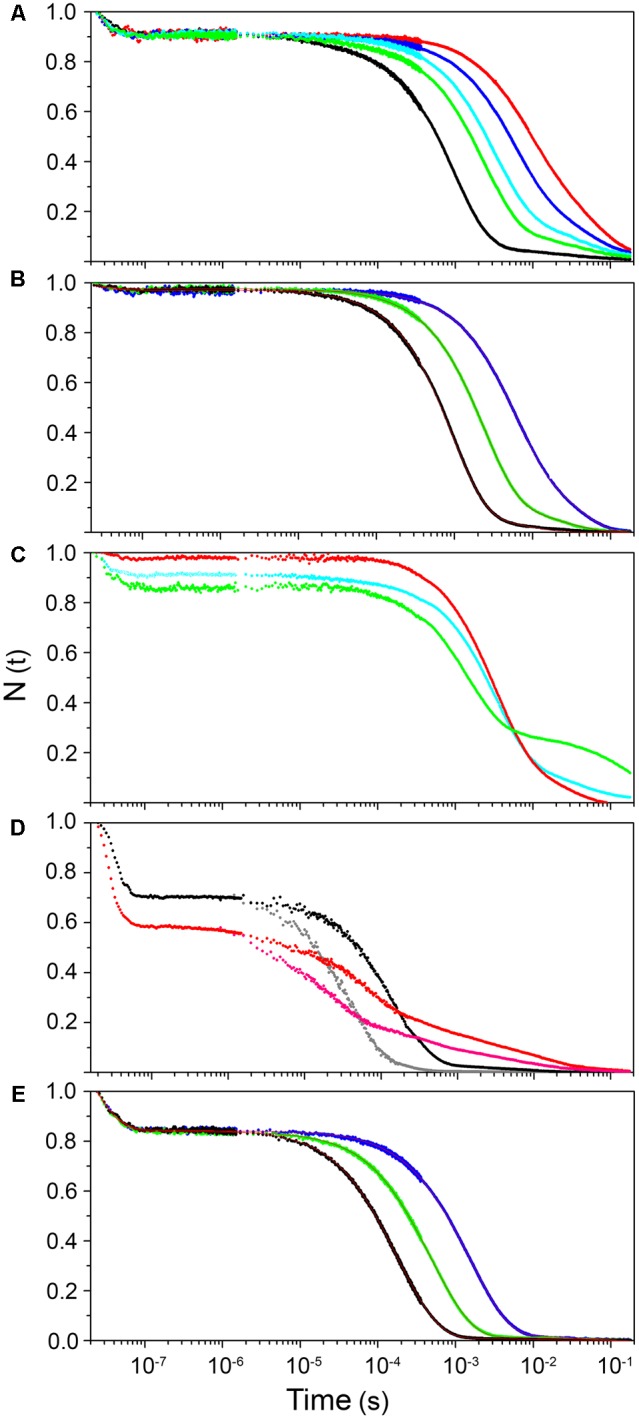
**Ligand-rebinding kinetics of LjGlbs and their mutant derivatives.** The figure shows CO-rebinding kinetics of the globins after photolysis at 532 nm and 20°C. The kinetics are reported as fractions of deoxy molecules and were calculated from the normalized absorption changes at 417 nm. All protein were used at a final concentration of 4 μM. **(A)** LjGlb1-1. [CO] = 800 μM (*black*), 300 μM (*green*), 200 μM (*cyan*), 100 μM (*blue*), and 50 μM (*red*). **(B)** LjGlb1-1 C8S. [CO] = 800 μM (*black*), 300 μM (*green*), and 100 μM (*blue*). Red solid lines are the best fits with a three-exponential decay function. **(C)** LjGlb1-1 C8S (*red*) and LjGlb1-1 C78S (*green*). [CO] = 200 μM. For comparison the rebinding kinetics to LjGlb1-1 at the same [CO] is also displayed (*cyan*). **(D)** LjGlb1-2 (*black and gray*) and LjGlb1-2 C79S (*red and magenta*). [CO] = 200 μM (*black and red*) and 800 μM (*gray and magenta*). **(E)** LjGlb2. [CO] = 800 μM (*black*), 300 μM (*green*), and 100 μM (*blue*). Red solid lines are the best fits with a four-exponential decay function.

As evidenced from the RR experiments (Supplementary Figure S3), the Fe-CO bond in hemeproteins is photolabile. LFP exploits this property to photodissociate the ligand with a short (nanosecond) laser pulse and monitor rebinding through the concomitant absorption changes. Photodissociated ligands can either be rebound by the heme from temporary docking sites within the protein matrix (geminate rebinding) or migrate to the solvent and be rebound at later times (bimolecular rebinding) (Abbruzzetti et al., 2006). The CO rebinding kinetics of LjGlb1-1, LjGlb1-2, and LjGlb2 were examined by LFP (**Figure 3**). For LjGlb1-1, a geminate phase was observed in the nanosecond range, which accounts for ∼10% of the rebinding and is well described by a single exponential relaxation (**Figure 3A**). On longer time scales, the progress curve for LjGlb1-1 is dominated by a large microsecond to millisecond phase with two easily recognizable kinetic steps (**Figure 3A**). The faster of these two steps has a clear bimolecular nature as demonstrated by the response of the kinetics to ligand concentration. When [CO] is decreased, the apparent rate of the faster step becomes lower, as expected for a diffusion-mediated bimolecular reaction. Accordingly, this step is identified as the reaction between CO and the LjGlb1-1 5c species. On the other hand, the amplitude of the slower step becomes higher when [CO] is decreased, whereas the apparent decay rate is unaffected. This is consistent with the transient formation of the bis-histidyl 6c species, which is observed when the distal His(E7) is coordinated to the sixth coordination site at the heme Fe, made available by the photolysis of the parent CO adduct. At lower [CO], the slower bimolecular rebinding to the 5c heme allows for a more efficient relaxation toward the bis-histidyl 6c species, thus resulting in a larger accumulation of this intermediate. Eventually, His(E7) will be displaced by CO, with a rate which is independent of [CO] and coincides with the His dissociation rate.

The overall kinetics is well described by the sum of three exponential decays, corresponding to the kinetic phases described above, and which are, in order of increasing lifetime: geminate rebinding, bimolecular binding to 5c species, and decay of bis-histidyl 6c species.

$$\begin{array}{c} HbCO\overset{hv}{\underset{k_{-1}}{⇄}}Hb:CO\overset{k_{2}}{\underset{k_{-2}}{⇄}}Hb+CO\overset{k_{\mathrm{on},H}}{\underset{k_{\mathrm{off},H}}{⇄}}Hb_{h}+CO \end{array}$$

The above scheme summarizes the relevant kinetic steps and the corresponding microscopic rate constants. After photodissociation with a photon of energy *hν*, the ligand can be rebound geminately from positions located within the protein matrix (Hb:CO) with rate k_-1_, or escape to the solvent (Hb_p_ + CO) with rate k_2_. Ligands can be rebound from the solvent with rate k_-2_. On the same time scale, the distal His can coordinate the Fe atom by forming a bis-histidyl hexacoordinate species Hb_h_ with rate k_on,H_. This species eventually decays with rate k_off,H_.

The bimolecular CO rebinding rate to the 5c protein, k_on,CO_, is related to microscopic rate constants according to this equation:

(1) $$\begin{array}{c} k_{\mathrm{on},\mathrm{CO}}\;=\;k_{-2}\frac{k_{-1}}{k_{-1}\;+\;k_{2}} \end{array}$$

The fitting, performed on a set of rebinding traces comprising five different CO concentrations, afforded to calculate apparent rates for each process. As expected, no difference in amplitude or apparent rate was observed for the geminate phase. On the contrary, the apparent rate for the bimolecular reaction between CO and the 5c heme increases linearly with [CO], and from the slope we could estimate the k_on,CO_ for the process (**Table 1**). The slowest process affords an estimate for the k_off,H_ rate. The two Cys residues of LjGlb1-1 appear to play a role in the overall structure and dynamics of the protein, with functional consequences on the heme ligand-binding kinetics (**Figures 3B,C**). Moreover, the C8S and C78S mutations induce very different effects on the overall CO rebinding kinetics. The C8S mutant is characterized by a smaller geminate amplitude (4%) with a minor decrease in the corresponding rate. The *k*_on,CO_ and the *k*_off,H_ are not substantially affected, whereas the extent of 6c species becomes a bit smaller, indicating a lower binding rate for His(E7). On the contrary, the C78S mutation leads to larger geminate recombination (13%), faster *k*_on,CO_, and slower *k*_off,H_. The larger accumulation of bis-histidyl species also indicates a larger *k*_on,H_. The changes in geminate recombination of the C8S and C78S mutants suggest that the egression pathway is affected by mutations at these two residues, albeit in the opposite direction.

The CO rebinding kinetics of LjGlb1-2 (**Figure 3D**) shares some similarity to that of LjGlb1-1, with a nanosecond geminate recombination followed by a biphasic kinetics. Thus, it is possible to recognize a bimolecular step corresponding to CO rebinding to a 5c species and a slower step associated with the decay of the bis-histidyl species. However, amplitudes and rates are dramatically different from the ones determined for LjGlb1-1. Geminate rebinding to LjGlb1-2 is a prominent process accounting for ∼30% of the kinetics, with an apparent rate which is not substantially different from that determined for LjGlb1-1. A minor contribution from a second transient in the bimolecular phase is detected in all traces for LjGlb1-2. Given the small amplitude and the irregular trend, it is ascribed to an impurity and neglected in the current analysis. The apparent rates for the bimolecular phase are clearly higher for this globin, as can be easily appreciated by visual inspection of the traces corresponding to the same [CO] (compare **Figures 3A,D**). The decay of the bis-histidyl species also appears faster than for LjGlb1-1. The kinetics are well reproduced by a sum of three exponential decays at all investigated values of [CO]. The fitting parameters reported in **Table 1** reflect the above qualitative description. The amplitude of geminate rebinding for LjGlb1-2 increases to ∼30%, indicating that for photodissociated ligands it is more difficult to reach the solvent than in the case of LjGlb1-1. A much higher value for *k*_on,CO_ is observed, in keeping with the higher geminate rebinding, and the decay of the bis-histidyl species is also a faster process. The C79S mutation has profound consequences on the rebinding kinetics (**Figure 3D**), with higher geminate rebinding, faster bimolecular rebinding, and accumulation of a much higher population of the bis-histidyl 6c species. Accordingly, the marker band of 5cHS in the RR spectrum decreased for this mutant (**Figure 1E**). **Table 1** shows that the amplitude of geminate rebinding increases to 46%, and that *k*_on,CO_ undergoes a twofold increase. The decay rate of the bis-histidyl 6c species is not substantially affected. Because this species is accumulated in higher yield, it is expected that the on-rate for the process will be higher.

The progress curve for CO rebinding to LjGlb2 shows the general kinetic pattern already highlighted for the class 1 nsHbs previously discussed, and is well described by a sum of exponential decays (**Figure 3E**). However, unlike the other globins described in this work, two exponential decays are needed to properly account for the bimolecular phase, a fact that may arise from two conformations coexisting in equilibrium. Their *k*_on,CO_ values differ by about twofold (**Table 1**). Although the long time tail of the rebinding kinetics is barely appreciable in the plot of **Figure 3E**, this kinetic phase has the typical features of the decay of bis-histidyl 6c species. The small amount of this intermediate is indicative of a very low, but not negligible, binding rate for the distal His.

Stopped flow, rapid mixing experiments were conducted with all nsHbs to determine the *k*_on,H_ and *k*_off,H_ rates. When deoxyferrous Hb solutions are mixed with a solution equilibrated with CO, the exogenous ligand is bound by the protein in a bimolecular reaction. In the 6c proteins like nsHbs, however, the endogenous ligand His(E7) must first dissociate from the heme so that the diatomic gas is able to bind to the heme. At high enough [CO], this step becomes rate limiting. For Hbs that are only partly hexacoordinated (in which a fraction of 5c species is present at equilibrium), binding of CO in the rapid mixing experiments is a biexponential process described by the following equation:

(2) $$\begin{array}{c} \Delta A_{\mathrm{obs}}\;=\;-A_{T}\left(F_{P}e^{-k_{on,CO}[\mathrm{CO}]t}\;+\;F_{H}e^{-k_{\mathrm{obs}}[\mathrm{CO}]t}\right) \end{array}$$

In this equation, Δ*A*_obs_ is the observed parameter for binding; *k*_on,CO_ is the bimolecular rate constant for CO binding to the 5c species; *k*_obs_ is the observed rate constant for binding following mixing; *F_P_* and *F_H_* are the fractions of protein in the 5c and 6c states; and *A_T_* is the total change in absorbance expected for the reaction, determined independently from ligand-free and ligand-bound absorbance spectra (Smagghe et al., 2006). The equation that describes the apparent rate *k*_obs_ for these kinetics is as follows (Trent et al., 2001):

(3) $$\begin{array}{c} k_{\mathrm{obs}}\;=\;\frac{k_{\mathrm{off},H}k_{\mathrm{on},\mathrm{CO}}\left[\mathrm{CO}\right]}{k_{\mathrm{on},H}\;+\;k_{\mathrm{off},H}\;+\;k_{\mathrm{on},\mathrm{CO}}[\mathrm{CO}]} \end{array}$$

**FIGURE 4:**
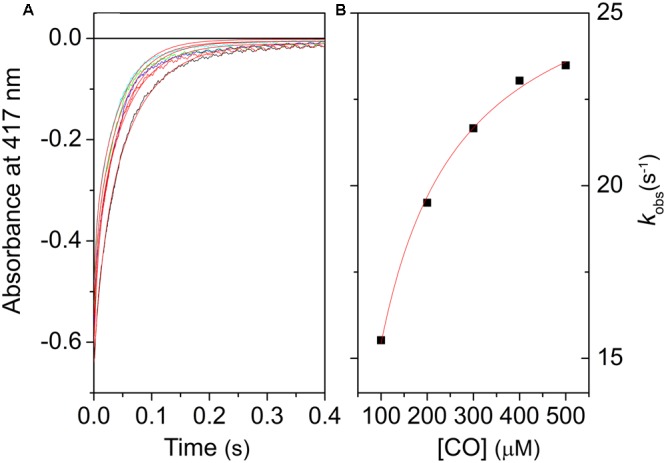
**Kinetics of CO binding to LjGlb1-1 observed after stopped-flow rapid mixing. (A)** Absorbance changes at 414 nm following rapid mixing of deoxyferrous LjGlb1-1 in CO-equilibrated buffer. Final [CO] after mixing was 100 μM (*black*), 200 μM (*red*), 300 μM (*green*), 400 μM (*blue*), and 500 μM (*cyan*). Red solid curves are the best fits with double exponential relaxations. **(B)** Observed rate constants for the slow kinetic phase of LjGlb1-1 as a function of [CO]. The rates of the fast kinetic phase exhibit a linear trend with slope *k*_on,CO_ (not shown in the figure). The red solid line is the best fit of the data with Eq. 2.

**Figure 4A** shows the progress curves for CO binding to LjGlb1-1 at several values of [CO], along with fits using double exponential relaxations. **Figure 4B** reports the [CO] dependence of the apparent rate constant associated with CO binding to the 6c species. Like other 6c globins, a typical trend is observed for the slow kinetic phase, where the apparent rate constant reaches a saturating value at high [CO] (Smagghe et al., 2006). This limiting value corresponds to the *k*_off,H_ rate. For all the proteins, the trend of the rates *k*_obs_ with [CO] is well described by Eq. 2, where *k*_on,H_ and *k*_off,H_ are held as free parameters, whereas the value of *k*_on,CO_, determined from flash photolysis, is held as a fixed parameter. The retrieved parameters for LjGlb1-1 and the other proteins are reported in **Table 1**.

**FIGURE 5:**
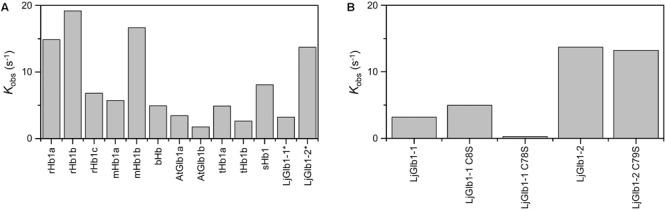
**Apparent binding rates of CO to several plant nsHbs. (A)** Comparison of apparent rates (*k*_obs_) determined from the values of *k*_on,CO_, *k*_on,H_, and *k*_off,H_ reported in the literature. [CO] = 10 μM. Abbreviations and references: rice Hb1 (rHb1a; Arredondo-Peter et al., 1997; Bisht et al., 2011), rice Hb1 (rHb1b; Smagghe et al., 2006), maize Hb1 (mHb1a; Smagghe et al., 2006), maize Hb1b (mHb1b; Smagghe et al., 2006), barley Hb1 (bHb1; Duff et al., 1997), *Arabidopsis thaliana* Glb1 (AtGlb1; Bruno et al., 2007a; Smagghe et al., 2009), tomato Hb1 (tHb1; Ioanitescu et al., 2005; Smagghe et al., 2009), soybean Hb1 (sHb1; Smagghe et al., 2009). Values of LjGlb1-1 and LjGlb1-2 (marked with asterisks) were obtained for this work. For rice and maize, values of two different class 1 Hbs, termed “a” and “b,” are provided. **(B)** Comparison of *k*_obs_ values of LjGlb1-1, LjGlb1-2, and their mutant derivatives.

**Figure 5A** compares the expected values for *k*_obs_ using the model gas CO for several nsHbs. It is quite clear that, due to the combination of rates *k*_on,CO_, *k*_on,H_, and *k*_off,H_, LjGlb1-2 binds CO with higher rate than LjGlb1-1 in these conditions. The *k*_obs_ values for LjGlb1-2 and its C79S mutant (**Figure 5B**) are quite similar. On the contrary, a fivefold decrease in the rate is observed for the C78S mutant of LjGlb1-1, which suggests a critical role of this residue in determining the rate constants relevant for *k*_obs_. In contrast, the effect of the C8S mutation appears to be negligible.

The values of the equilibrium constants (*K*_H_) for the binding of the distal His(E7) to the hemes of the three LjGlbs are shown in **Table 1**. The *K*_H_ of LjGlb1-1 demonstrates that a fraction of 5c species is present at equilibrium. This is in keeping with the presence of a small intensity, second ν_3_ band observed at 1467–1475 cm^-1^ in the RR spectrum (**Figure 1A**), indicating the presence of a 5cHS ferrous form. The LjGlb1-1 C8S mutation slightly shifts the equilibrium toward the 5cHS species. The opposite effect is observed for the LjGlb1-1 C78S mutant. The weak second ν_3_ band observed at 1467–1475 cm^-1^ appears to behave consistently (**Figures 1B,C**). The *K*_H_ of LjGlb1-2 clearly indicates that the unliganded ferrous form of the protein is mostly present as the bis-histidyl 6c species, as anticipated by the absorption spectra (Sainz et al., 2013) and the RR spectra (**Figure 1**). The LjGlb1-2 C79S mutation results in heterogeneous kinetics in the time range where the bis-histidyl species is formed and decays, where two exponential decays are needed to account for the measured time course of CO binding. Plotting the two rate constants as a function of [CO] and fitting their trend using Eq. 2 allows to retrieve the *k*_on,H_ and *k*_off,H_ values reported in **Table 1**. For both conformations, these rates result in stronger hexacoordination than observed for the wild-type protein. The reason for the presence of the two species is as yet unclear. Finally, the *K*_H_ of LjGlb2 is quite low, indicating a substantially lower stability of the bis-histidyl 6c species. Consistently, RR spectra of LjGlb2 (**Figure 1**) show a remarkably high population of 5cHS species, and the absorption spectra clearly show a mixture of 5c and 6c species (Sainz et al., 2013).

### Nitric Oxide Dioxygenase Activities

**FIGURE 6:**
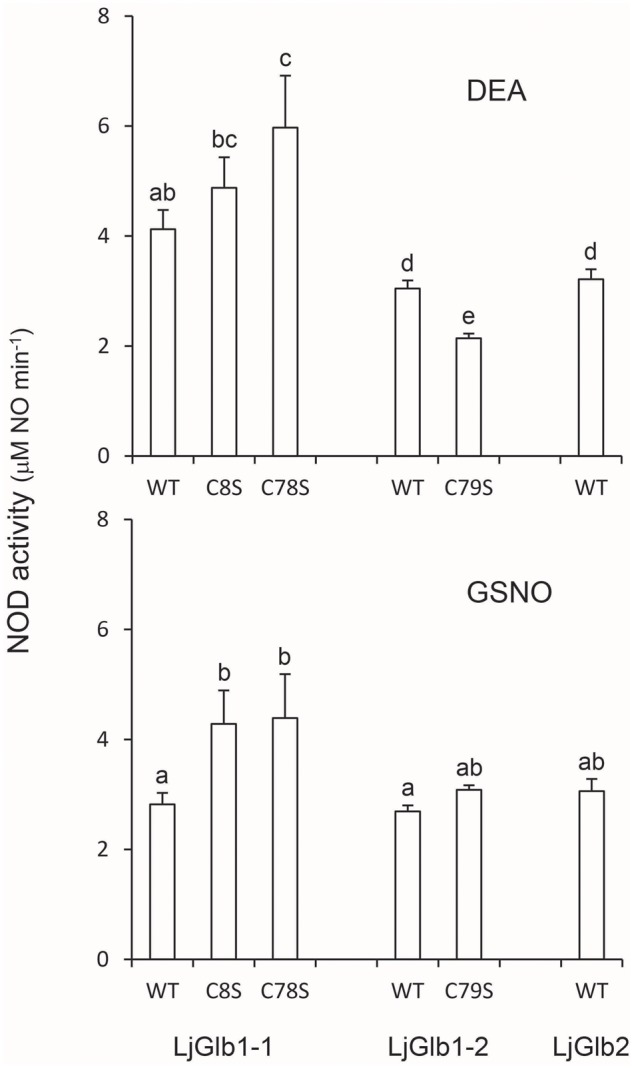
**NOD activities of LjGlbs and some of their mutated forms.** The activities were measured with DEA or GSNO as NO donors. Values are means ± SE of at two to three independent protein preparations. Means denoted by the same letter were not statistically different (*P* < 0.05) based on the Duncan’s multiple range test.

Because both class 1 and class 2 nsHbs are able to scavenge NO *in vitro* and *in vivo* (Perazzolli et al., 2004; Igamberdiev et al., 2006; Hebelstrup and Jensen, 2008; Smagghe et al., 2008), we measured NOD activities of LjGlbs, as well as of some of their mutated forms, to identify possible differences among the proteins and to determine whether Cys residues play a role in NO scavenging activity. To this end, we used a well-known artificial NO donor (DEA) and a physiological NO donor (GSNO). At the concentrations employed, both compounds released NO linearly for ∼4 min, at which time NO concentration stabilized. The oxyferrous Hbs were then added and the initial rate of NO consumption was measured and expressed on the basis of hemeprotein concentration (**Figure 6**). The decrease in NO concentration was due to NOD activity mediated by the hemes and was unrelated to scavenging by Cys residues because the respective ferric Hbs had no activity. We found that wild-type LjGlb1-1, LjGlb1-2, and LjGlb2 exhibited similar NOD activities regardless of the NO donor. Also, there were no major differences of NOD activity between the wild-type and the mutated proteins, although the LjGlb1-1 mutants displayed higher NOD activity than the wild-type LjGlb1-1. The cause for this minor, yet significant, increase is uncertain because it did not occur in LjGlb1-2 C79S (**Figure 6**), which again indicates that the Cys residues are not involved in NO scavenging.

## Discussion

In this work, the electronic and ligand binding properties of the heme environments of the three nsHbs of *L. japonicus* were examined by combining several spectroscopies and assaying the NOD activities of the proteins. The comparisons between LjGlb1-1 and LjGlb1-2, as well as between class 1 and class 2 nsHbs, were facilitated by using mutated proteins, which enabled us to determine the effect of Cys residues on protein stability and ligand affinity. This type of studies on plant nsHbs is scarce, yet important to understand protein function.

Although the general behavior of the two class 1 nsHbs is similar, the details of their distal pocket and the ligand rebinding kinetics show significant differences. For LjGlb1-1, the geminate phase of ligand binding is comparable to that of other class 1 nsHbs. Thus, the CO rebinding to rice Hb1 is characterized by ∼10% geminate rebinding that occurs with a nearly mono-exponential kinetics. Similarly, the geminate rebinding to AtGlb1 is a process with a comparable amplitude, but a slightly more complex kinetics that is well described by a bi-exponential relaxation (Abbruzzetti et al., 2006). In AtGlb1 the bi-exponential nature of the kinetics was interpreted as a result of the migration of the photodissociated ligand to nearby cavities, from which the ligand is rebound at later times with different rates (Abbruzzetti et al., 2007; Bruno et al., 2007a). Both the equilibrium binding constants and the binding rates to hemeproteins are profoundly influenced by structural properties of the active site, including the presence of temporary docking sites within the protein matrix, and tunnels connecting the interior of the protein with the solvent. The strong interaction of the CO ligand with the distal His residue observed for LjGlb1-1 and its C8S and C78S mutants, for which the ν_Fe-CO_ modes were found at ∼533–536 cm^-1^ and δ_Fe-CO_ at 587–589 cm^-1^ (**Figure 2** and Supplementary Figure S3), may be taken as the main reason for the high affinity of this protein for diatomic gases, because this interaction is expected to decrease substantially the ligand dissociation rate constant. Notably, the same values (535 cm^-1^) were found for barley Hb1 (Das et al., 1999) and AtGlb1 (Bruno et al., 2007a). The *K*_H_ value of LjGlb1-1 is consistent with those of other class 1 nsHbs (Supplementary Figure S4A). It is interesting to note that while *k*_on,H_ and *k*_off,H_ show a large variability across the class 1 nsHbs, these rate constants appear to be strongly correlated (*r* = 0.87; Supplementary Figure S4B). The slope provides a *K*_H_ of 1.6 ± 0.2, a value that implies partial hexacoordination for the equilibrium, ligand-free deoxyferrous species. A similar average value of 1.7 ± 0.2, estimated from the analysis of several class 1 nsHbs, was reported earlier (Smagghe et al., 2009). The reasons for the correlation may be ascribed to the dynamics of the different proteins and may involve an enthalpy–entropy compensation. The transition from the bis-histidyl hexacoordination to pentacoordination implies conformational changes of the protein. Central to this conformational change is the peculiar translation of helix E along its axis. The flexibility of the CD and EF loop regions in class 1 nsHbs allows the piston motion of the E-helix that accompanies dissociation of the distal His and subsequent ligand binding. This flexibility, along with the unfavorable interaction of Phe(B10) with the coordinated distal His, promotes reversible hexacoordination (Hoy et al., 2007).

LjGlb1-2 is clearly an outlier with respect to CO binding rate, His binding and dissociation rates, and *K*_H_ values, which are much higher than the typical values observed for class 1 nsHbs. The amplitude of geminate rebinding for LjGlb1-2 is much larger than the typical values reported for other class 1 nsHbs and, in fact, it is similar to the amplitude observed for AtGlb2 (Bruno et al., 2007a,b). Likewise, the *k*_on,CO_ of LjGlb1-2 resembles the values of class 2 nsHbs rather than those of class 1 nsHbs (Supplementary Figure S5). The distal pocket of LjGlb1-2 is quite closed, with ν_Fe-CO_ mode at 519 cm^-1^, suggestive of a strong interaction with distal pocket residues, but this interaction is less strong than for LjGlb1-1. Unlike the case of LjGlb1-1, the presence of a closed distal pocket in LjGlb1-2 does not impair geminate recombination, which occurs with a remarkably large amplitude (∼30%), probably due to the weaker interaction. The ν_Fe-CO_ mode undergoes a dramatic change for the LjGlb1-2 C79S mutant, shifting to 492 cm^-1^ (**Figure 2E**), a value indicative of an open heme pocket. Consistently, the amplitude of the geminate recombination becomes ∼50%, showing that the barrier encountered by the ligand is further decreased. For LjGlb2, the ν_Fe-CO_ mode is similar to that observed for CO-ligated mammalian Mbs at neutral pH (Sage et al., 1991). The prevalence of the 5c heme in the deoxy LjGlb2 seems to indicate that, for this globin, the heme-pocket region is indeed more Mb-like.

Plant nsHbs contain Phe(B10) and His(E7) in their distal pockets (Arredondo-Peter et al., 1997; Hoy et al., 2007). These residues are crucial for protein function because they strongly modulate ligand binding to the heme. The distal His residue in LjGlb1-1 and LjGlb1-2 may also impose a barrier to rebinding and favor ligand escape to the solvent through the protein matrix, thus resulting in small amplitude geminate rebinding. The LjGlb1-1 C8S and C78S mutations do not change substantially the distal pocket interactions of the bound CO, whereas the geminate recombination is changed, suggesting that the distal pocket is somehow perturbed. However, the two mutations appear to lead to opposite effects, as was also the case for the *K*_H_ values.

The measurements of NOD activities complemented our spectroscopic studies because NO is a typical ligand of plant and animal Hbs (Brunori et al., 2005; Igamberdiev et al., 2006; Smagghe et al., 2008). Our results demonstrate that both class 1 and class 2 nsHbs are able to scavenge NO at similarly high rates (**Figure 6**), comparable to those reported for other Hbs using a different assay system (Smagghe et al., 2008). The initial rates of NO consumption measured here are due to genuine NOD activity catalyzed by the hemes and not to Cys nitrosylation because ferric Hbs had no activity and because the wild-type and mutant proteins displayed similar NOD activities. These observations are consistent with the finding that barley Hb1 and its single Cys mutant also showed similar NOD activities (Bykova et al., 2006) and with the proposal that NOD activities are widespread amongst plant and animal Hbs (Smagghe et al., 2008; Gardner, 2012). Interestingly, the NOD activity of LjGlb1-1 would provide the only way, to our knowledge, for this protein to remove bound O_2_ because it has an extremely high O_2_ affinity (*K*_O_2__ = 50 pM) due to a very slow dissociation rate (*k*_off_ = 0.004 s^-1^) (Sainz et al., 2013). Because NOD activity yields ferric Hb, enzymatic and/or non-enzymatic systems are required to regenerate oxyferrous Hb and sustain NOD activity *in vivo*. These may include free flavins and flavoproteins (Becana and Klucas, 1992; Igamberdiev et al., 2006; Sanz-Luque et al., 2015). Further studies on the identification of ferric Hb reducing mechanisms shall shed light on this controversial issue (Smagghe et al., 2008).

Our results may be useful for agrobiotechnological applications and for legume researchers in general because they reveal distinct biochemical properties not only between class 1 and class 2 nsHbs, but also between the two members of the same class. The differences in CO binding kinetics found in this work, along with the large variations in O_2_ affinities and expression profiles reported earlier (Sainz et al., 2013), strongly suggest that the three proteins perform non-redundant functions. Overexpression of LjGlb1-1 increases the symbiotic performance of *L. japonicus* (Shimoda et al., 2009) and, conversely, the knocked-out line shows alterations in the infection process and produces fewer nodules than the wild-type line (Fukudome et al., 2016). Consequently, transgenic approaches aimed at increasing the content of each of the three nsHbs, or of a combination of them, in the model legume *L. japonicus* (first stage) and in a crop legume with comparable nsHbs, such as soybean or common bean (second stage), are likely to result in outperformance of plants, at least under symbiotic conditions. Likewise, the three genes may be successfully implemented in plant breeding programs because overexpression of class 1 nsHbs improves survival of hypoxic stress in *A. thaliana* (Hunt et al., 2002) and maize (Mira et al., 2016), and therefore have the potential of conferring tolerance to abiotic stresses.

## Conclusion

Spectroscopic analyses of LjGlbs reveal major differences between the two phylogenetic classes of nsHbs and also between the two members of the same class, strongly suggesting that the three globins perform non-redundant functions. Specifically, the degree of binding of the distal His(E7) to the heme iron in the deoxyferrous state greatly differs among the LjGlbs studied here. Whereas the equilibrium constant for His binding (*K*_H_) of LjGlb1-1 is in line with those determined for other class 1 nsHbs (Smagghe et al., 2009), LjGlb1-2 behaves more like class 2 nsHbs, showing complete bis-histidyl hexacoordination in the deoxyferrous state. Moreover, LjGlb2 is an atypical class 2 nsHb because it is mostly pentacoordinate in the deoxyferrous form. Upon CO ligation, the bound CO is very strongly stabilized by hydrogen bonding to nearby amino acid residues, probably His(E7), in LjGlb1-1 and its C8S and C78S mutants, but the stabilization is less strong in LjGlb1-2 and LjGlb2. In the latter, the CO is similarly stabilized as in mammalian Mbs and Hbs, which are also pentacoordinate globins. The LjGlb1-1 C8S and C78S mutations caused changes in CO geminate recombination, indicating perturbations of the heme environment. Remarkably, the LjGlb1-2 C79S mutation removes the CO stabilization and gives rise to an open heme pocket. The CO stabilizations of the three globins are consistent with their O_2_ affinities (Sainz et al., 2013), following the order LjGlb1-1 > LjGlb1-2 > LjGlb2. Considering that the stronger the stabilization, the higher the affinity, we conclude that the affinities for diatomic ligands are essentially determined by the dissociation rate constants (*k*_off_). In contrast to the differences observed for CO binding and geminate recombination, the NOD activities of the three nsHbs were rather similar, which leads us to conclude that the activities are an intrinsic property of the hemes and that the small variations seen in the mutated proteins are due to alterations in the heme environment and not to direct NO scavenging by Cys residues.

## Author Contributions

All authors performed experiments. SA and SB interpreted results. BC, SVD, SD, CV, and MB interpreted results and wrote parts of the manuscript.

## Conflict of Interest Statement

The authors declare that the research was conducted in the absence of any commercial or financial relationships that could be construed as a potential conflict of interest.
