# Supplementary material for: Characterization of the Heme Pocket Structure and Ligand Binding Kinetics of Non-symbiotic Hemoglobins from the Model Legume Lotus japonicus
Source: Front Plant Sci. 2017 Apr 4;8:407. doi: 10.3389/fpls.2017.00407 (PMC5378813; doi:10.3389/fpls.2017.00407)
Supplement: Supplementary file 1 [file Image_1.PDF]

LjGlb1-1 C8S

MSTLGST**S**FTEEQEALVVKSWSVMKKNSAELGLKLFLKIFEIAPSAQKLFSFLRDSKVPLEENPKLKPHAMSVFVMT
CESAAQLRKAGKVTVRESTLKKLGATHYKYGVVNEHFEVTKFALLDTIKEAVPEMWSPEMKNAWAQAYDQLVGA
IKSEMKPSSS-

LjGlb1-1 C78S

MSTLGSTCFTEEQEALVVKSWSVMKKNSAELGLKLFLKIFEIAPSAQKLFSFLRDSKVPLEENPKLKPHAMSVFVMT
SESAAQLRKAGKVTVRESTLKKLGATHYKYGVVNEHFEVTKFALLDTIKEAVPEMWSPEMKNAWAQAYDQLVGA
IKSEMKPSSS-

LjGlb1-2 C79S

MAENTTTIAFTEEQEALVVKSWNAMKKDSAELSFKFFSKILEIAPPAKQLFSFLRDSEVPLDQNPKLKPHAMSVFLM
T**S**ESAAQLRKEGKVTVRESNLKKLGATHFKKGVIPEHFEVTKQALLDTIKEAVPELWSLELKDAWAIAHDQLASAIIA
EMKPES-

LjGlb2 C65S

MATFSEEQEALVNSSWEAFSQNIPQLSIIFYTSILEKAPEAKAMFSFLKDSDGVPKDNLDLEAH**S**EKVFELTRNSALQ
LRAKGKVEVERIALKFLGYVHAQRRVLDPHFLVLKEALLKTLKEAMGDKWSEEVSNAWGIAYDELAGVIKKGMS-

FIGURE S1 | Mutated versions of the LjGlbs. The Cys → Ser mutation is marked in red. The N-terminal poly-His tag is MRGSHHHHHHGMASMTGGQQMGRDLYDDDDKDHPFT for all of them.
