# Supplementary material for: Characterization of the Heme Pocket Structure and Ligand Binding Kinetics of Non-symbiotic Hemoglobins from the Model Legume Lotus japonicus
Source: Front Plant Sci. 2017 Apr 4;8:407. doi: 10.3389/fpls.2017.00407 (PMC5378813; doi:10.3389/fpls.2017.00407)
Supplement: Supplementary file 2 [file Image_2.PDF]

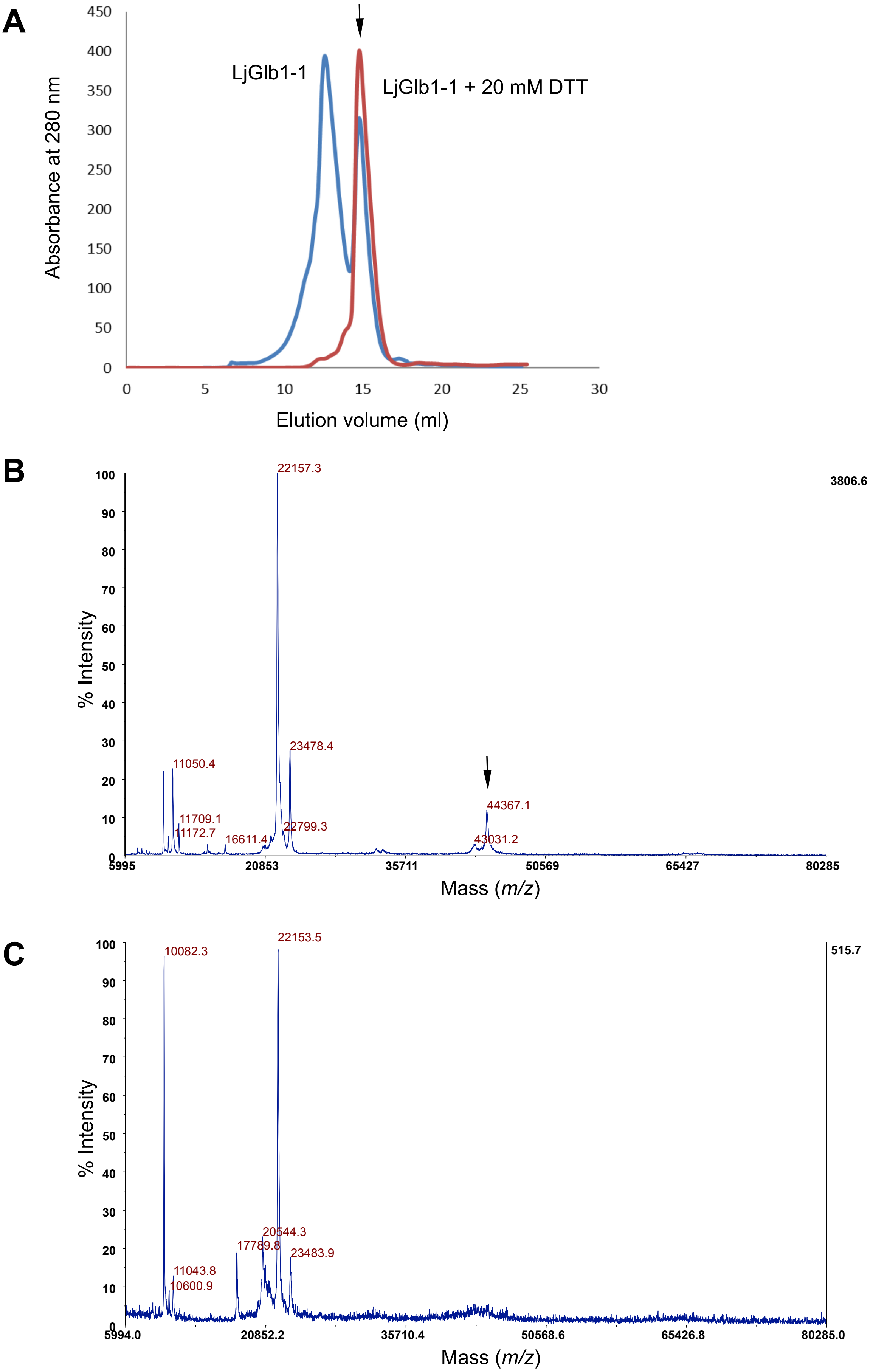

FIGURE S2 | The LjGlb1-1 protein can form homodimers through a disulfide bridge. (A) FPLC separation of the monomeric and dimeric forms. The dimer peak (*arrow*) disappears upon addition of 20 mM dithiothreitol (DTT). **(B)** Mass spectrometry analysis confirms the presence of the homodimer. **(C)** Mass spectrum showing that the dimer peak (*arrow*) disappears upon DTT addition.
