# Supplementary material for: Characterization of the Heme Pocket Structure and Ligand Binding Kinetics of Non-symbiotic Hemoglobins from the Model Legume Lotus japonicus
Source: Front Plant Sci. 2017 Apr 4;8:407. doi: 10.3389/fpls.2017.00407 (PMC5378813; doi:10.3389/fpls.2017.00407)
Supplement: Supplementary file 3 [file Image_3.pdf]

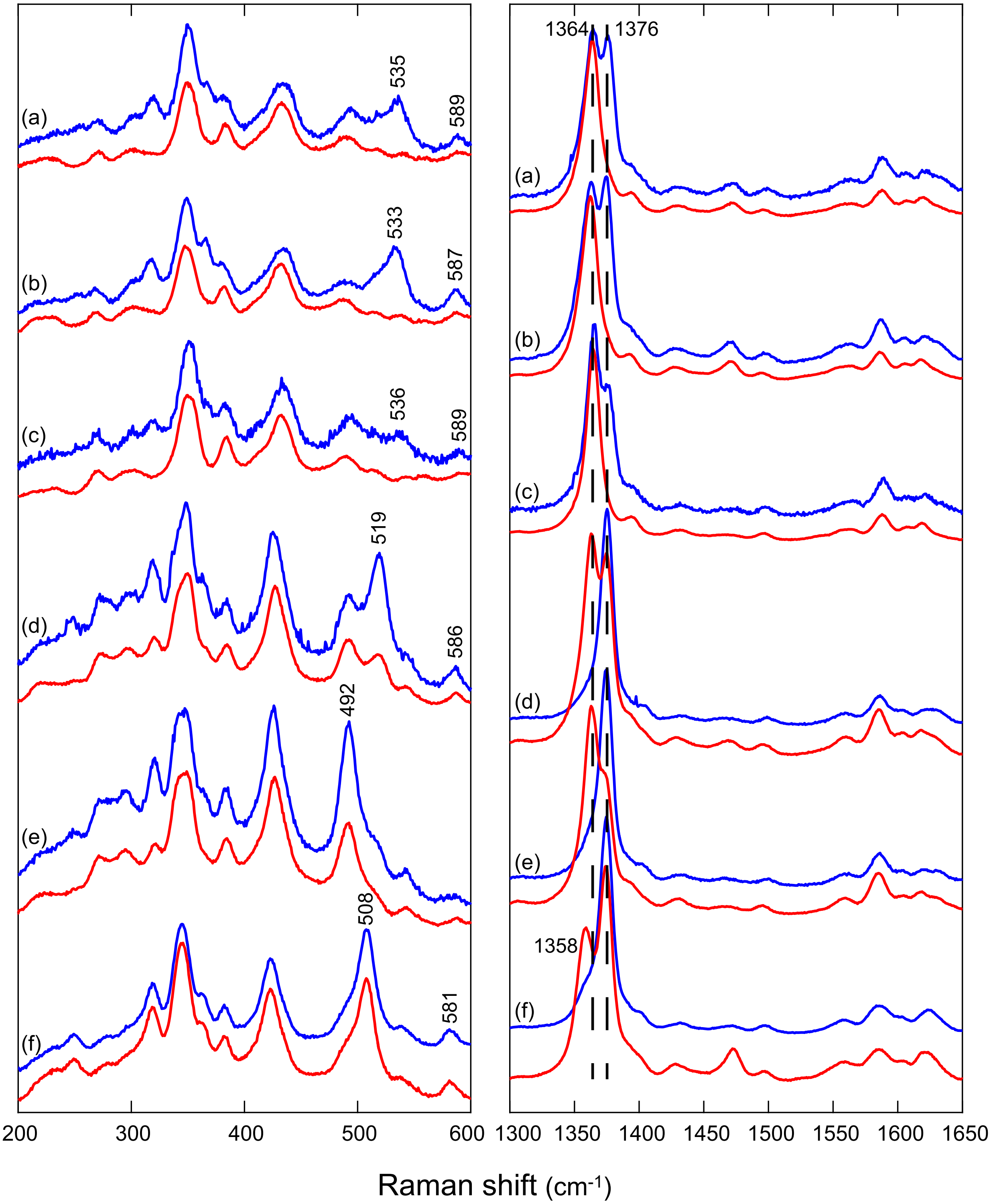

FIGURE S3 | RR spectra of LjGlbs in the ferrous CO-ligated form. The spectra correspond to (a) LjGlb1-1, (b) LjGlb1-1 C8S, (c) LjGlb1-1 C78S, (d) LjGlb1-2, (e) LjGlb1-2 C79S, and (f) LjGlb2. The upper spectra (*blue*) were recorded at low laser power (1 mW) and the lower spectra (*red*) at high laser power (35-165 mW).
