# Supplementary material for: Characterization of the Heme Pocket Structure and Ligand Binding Kinetics of Non-symbiotic Hemoglobins from the Model Legume Lotus japonicus
Source: Front Plant Sci. 2017 Apr 4;8:407. doi: 10.3389/fpls.2017.00407 (PMC5378813; doi:10.3389/fpls.2017.00407)
Supplement: Supplementary file 4 [file Image_4.PDF]

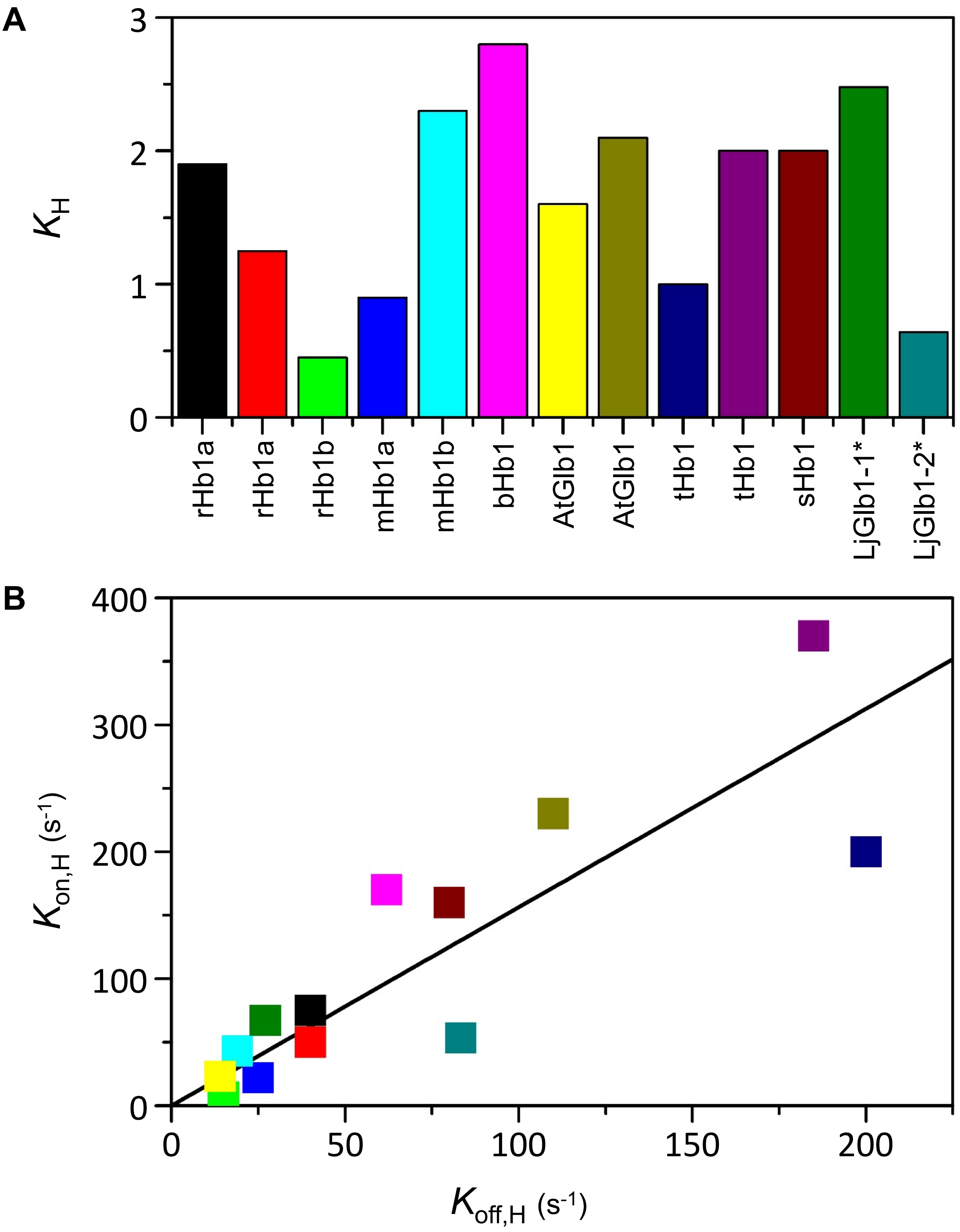

FIGURE S4 | Kinetics for the binding of the distal His(E7) to the heme in class 1 nsHbs. (A) Comparison between equilibrium constants (K_H) for binding of the distal His(E7) to the heme for several class 1 nsHbs. **(B)** Correlation between binding and dissociation rate constants for the distal His(E7). Abbreviations are the same as for Figure 5.
