# Supplementary material for: Characterization of the Heme Pocket Structure and Ligand Binding Kinetics of Non-symbiotic Hemoglobins from the Model Legume Lotus japonicus
Source: Front Plant Sci. 2017 Apr 4;8:407. doi: 10.3389/fpls.2017.00407 (PMC5378813; doi:10.3389/fpls.2017.00407)
Supplement: Supplementary file 5 [file Image_5.PDF]

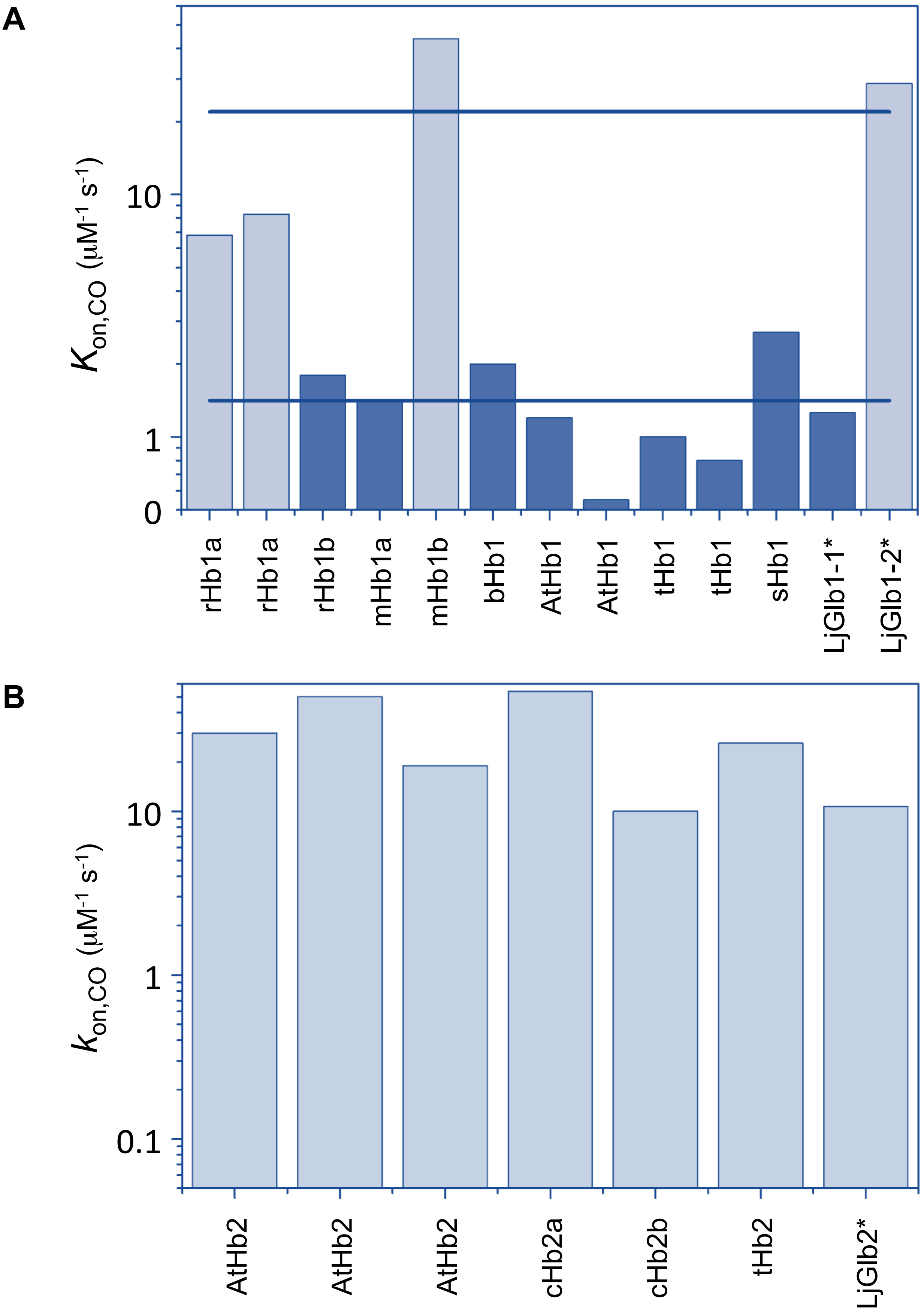

FIGURE S5 | Comparisons of $k_{on,co}$ for several class 1 and class 2 nsHbs. (A) Class 1 nsHbs. Dark blue and light blue bars distinguish between class 1 nsHbs with low and high $k_{on,CO}$ rates, respectively. Solid horizontal lines are average values for the two groups. Abbreviations and references are the same as for Figure 5. The LjGlb1-1 and LjGlb1-2 values (marked with asterisks) were obtained for this work. **(B)** Class 2 nsHbs. Abbreviations and references: *Arabidopsis thaliana* Glb2 (AtGlb2; Bruno et al., 2007a; Uzan et al., 2004; Smagghe et al., 2009), chicory Hb2a (cHb2a; Smagghe et al., 2009), chicory Hb2b (cHb2; Smagghe et al., 2009), tomato Hb2 (tHb2; Smagghe et al., 2009). The LjGlb2 value (marked with asterisk) was obtained for this work. For chicory Hb, values of two different class 1 Hbs, termed "a" and "b", are given.
